# Fine-Grained, Local Maps and Coarse, Global Representations Support Human Spatial Working Memory

**DOI:** 10.1371/journal.pone.0107969

**Published:** 2014-09-26

**Authors:** Mohammad Zia Ul Haq Katshu, Giovanni d'Avossa

**Affiliations:** 1 School of Psychology and Wolfson Centre for Clinical and Cognitive Neuroscience, Bangor University, Bangor, United Kingdom; 2 Division of Psychiatry and Applied Psychology, Institute of Mental Health, University of Nottingham, Nottingham, United Kingdom; Centre de Neuroscience Cognitive, France

## Abstract

While sensory processes are tuned to particular features, such as an object's specific location, color or orientation, visual working memory (vWM) is assumed to store information using representations, which generalize over a feature dimension. Additionally, current vWM models presume that different features or objects are stored independently. On the other hand, configurational effects, when observed, are supposed to mainly reflect encoding strategies. We show that the location of the target, relative to the display center and boundaries, and overall memory load influenced recall precision, indicating that, like sensory processes, capacity limited vWM resources are spatially tuned. When recalling one of three memory items the target distance from the display center was overestimated, similar to the error when only one item was memorized, but its distance from the memory items' average position was underestimated, showing that not only individual memory items' position, but also the global configuration of the memory array may be stored. Finally, presenting the non-target items at recall, consequently providing landmarks and configurational information, improved precision and accuracy of target recall. Similarly, when the non-target items were translated at recall, relative to their position in the initial display, a parallel displacement of the recalled target was observed. These findings suggest that fine-grained spatial information in vWM is represented in local maps whose resolution varies with distance from landmarks, such as the display center, while coarse representations are used to store the memory array configuration. Both these representations are updated at the time of recall.

## Introduction

Early students of cognition viewed the relation between memory and perception as analogous to that between a portrait and the scene portrayed [1], [2]. Moreover, a long-standing intellectual tradition has since held that all memories are, or can be, spatially organized, since imposing a spatial structure facilitates the maintenance and recall of information, whether visual or conceptual [2], [3]. While a prominent contemporary account of working memory has embraced this original metaphor of visual memory as a sketch of previously viewed scenes [4], [5], recent investigations examining the limits to the information that can be held in visual working memory (vWM), do not support a spatially based, analogical model of vWM [6]–[9]. Initially, the observations that the ability to detect changes between subsequently presented scenes degraded rapidly when the scenes contained more than three or four objects, regardless of their complexity, led to the suggestion that visual data are stored in a limited number of object specific slots, each slot endowed with unlimited resolution [6]. Later, this model was revised to account for the fact that recall of visual data shows decrements whenever more than one object is held in vWM. The revised model suggested instead that slots have limited resolution and when the number of objects held in memory is less than the number of slots, more than one slot is used to store the same object [7]. Improved recall precision can then be achieved by averaging over independent memory representations. An alternative interpretation of the gradual decline in recall precision with memory load is that limited resolution resources are used to represent specific visual dimensions, such as color, position and orientation [8], [9]. Consequently, as the number of features in a given dimension increases, a smaller fraction of the global resource is available to represent each feature. This model predicts no upper limit on the number of features, and consequently objects, that can be held in memory, but shares with the former model the assumption that memory resources are not tuned to specific features within a given dimension. These proposals imply that memory differs from sensory representations in visual cortex, which are tuned to specific features, such as the specific location, orientation or color [10]–[12].

A large body of neurophysiological work has indicated that during maintenance of information in vWM, sustained increases in neural activity take place in frontal and parietal areas, which are modulated by memory load [13]–[17]. The early slot model provided an elegant explanation of these findings, since the amplitude of the sustained neural activity appeared to track the number of slots utilized. However, neither the revised version of the slot model nor the resource model account for the effects of memory load on the amplitude of delay period neural activity, since both assume that memory utilizes all available slots or resources, irrespective of memory load. Interestingly, more recent fMRI data suggest that visual information can be decoded from spatial patterns of BOLD activity in early visual cortical areas, during the delay phase of vWM tasks, even though no overall increase in BOLD activity is observed there [18]–[22]. Considering that these cortical regions contain neurons with receptive fields that span limited areas of the visual field, the aforementioned fMRI findings suggest that capacity limitations in recalling the details of a memorized scene depend on spatially curtailed processes and hence that a target's position may affect the resolution of its memory representation.

Moreover, neither slots nor resource models, which assume that features belonging to different objects are stored independently of each other, account for the finding that recall of a specific feature not only depends on the value of that feature, but also on the values of other features of the same dimension within the memory array [23]–[25]. Further evidence for global effects in vWM is provided by the finding that neural responses in parietal regions of non-human primates, performing a match to sample task, are affected by the spatial configuration of the memory array, but are invariant to the position of the array in the visual field [26], [27], suggesting that higher order neurons update their spatial selectivity, to gain access to the configuration of the visual scene.

We examined how precision and accuracy of spatial recall depends on local factors, namely the location of the memory target, global factors, namely the overall configuration of the items held in memory and configurational information presented at recall. We found that recall precision depends not only on the number of items held in memory, as previously reported [6]–[9], but also on the target location, while recall accuracy depends on the overall spatial configuration of the memorized items. Moreover, presenting configurational information at recall affected both the accuracy and precision of recall. We propose that spatial information is maintained in both local, variable resolution spatial maps, and coarse representations of the overall configuration of the memory items and that both representations are updated at the time of recall.

## Results

### Target location and memory load affect spatial recall precision

To characterize spatial recall performance, the systematic and variable components of the recall errors were quantified separately. The systematic error is the component that is consistently repeated over trials, while the variable error is the component whose value changes unpredictably trial by trial. The terms recall ‘accuracy’ and ‘precision’ are used throughout this paper to refer to the reciprocal of the magnitude of the systematic and variable errors, respectively.

**Figure 1:**
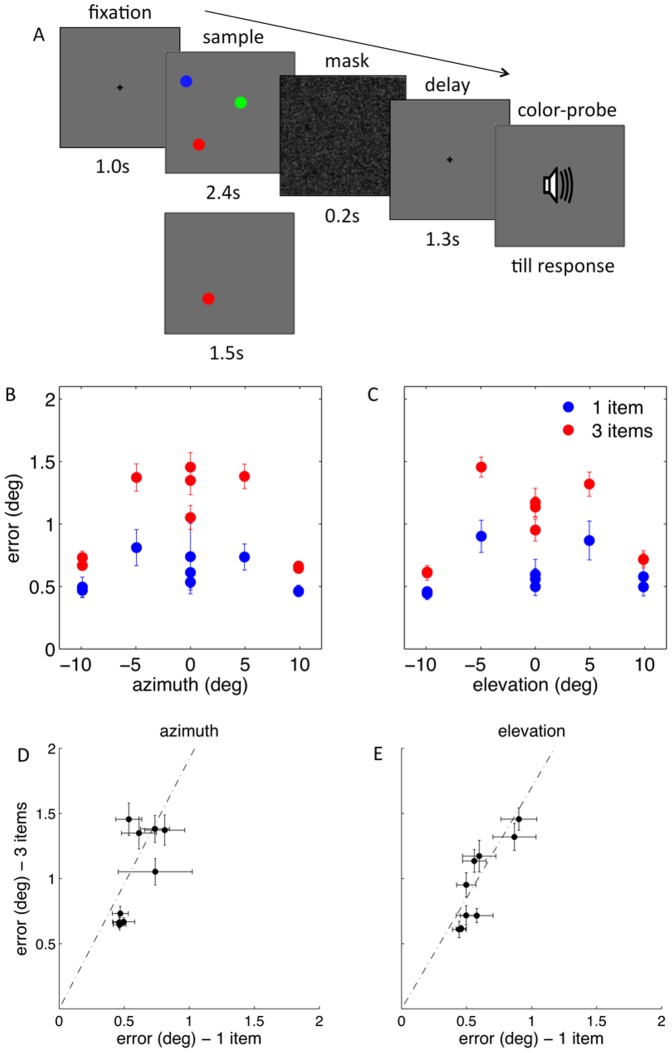
Memory load and target location affect spatial recall. (A) Participants memorized the location of either one or three items. After a pattern mask and blank interval, the target to be recalled was indicated by its color. (B) The standard deviation of the recall error is shown as a function of target azimuth, when the memory load is one (in blue) and three (in red). (C) The recall error as a function of target elevation. The variable error is smaller for targets closer to the center and boundaries of the display compared to intermediate positions. (D) The standard deviation of the recall error when memory load was three is shown as a function of the error standard deviation when memory load was one for target azimuth, and (E) elevation. Each point represents the group averaged error standard deviation at one of the nine target locations. The vertical and horizontal error bars are standard errors of the mean. The dash-dot line represents the group average best fitting multiplicative model.

We measured the precision in recalling the position of simple colored discs, when one or three were presented in the sample display (Figure 1A). Figures 1B and C show that the target location affected the standard deviation of the variable error of spatial recall. Specifically, targets located between the center and the boundaries of the display, were recalled less precisely than targets close to either the center or the boundaries of the display, suggesting that proximity to stable landmarks may facilitate the encoding and recall of spatial data in vWM. Moreover, the effect of target location on spatial recall was qualitatively similar whether the participants memorized one or three items. However, memory load did change the overall recall precision, which was diminished when the participants had to remember three rather than one item. Two-way, repeated-measures ANOVAs confirmed that the variance of the recall error was affected by the target position along horizontal axis, namely target azimuth - F(8,56) = 16.46, p<0.001; the target position along vertical axis, namely target elevation - F(8,56) = 29. 18, p<0.001), and memory load (for target azimuth - F(1,7) = 30.61, p = 0.001; for target elevation - F(1,7) = 25.44, p = 0.001). Interestingly, there was a significant interaction between target location and memory load (azimuth - F(8,56) = 8.29, p<0.001; elevation - F(8,56) = 8.33, p<0.001), strongly suggesting that the effects of target location and memory load on recall precision are not independent. This result is important since it is at odds with the possibility that the effects of target location and memory load on recall arise at separate stages. For example, a plausible hypothesis could have been that memory load effects reflect the limited capacity of working memory, while those of target location, the spatiotopic organization of early perceptual mechanisms. However, if this were the case, the effects on recall variance of target location would be additive with those of memory load, which is contrary to the finding reported above. Figures 1D and E show, for each target location, the group averaged recall error's standard deviation, when memory load was three, as a function of the standard deviation of the error, when memory load was one. The relation between the standard deviations is multiplicative rather than additive. We estimated, participant by participant, the best fitting additive and multiplicative models. The log-likelihood of the multiplicative model was greater than the log-likelihood of the additive model in all participants (azimuth - t(7) = 3.11, p = 0.017; elevation - t(7) = 3.10, p = 0.017), except one, in whom the effects of memory load were least prominent. Moreover, we found that the error standard deviation at each of the target locations was proportional to the square root of the memory load. In fact, the recall error standard deviation, when observers memorized three items, was 1.87 (95% CI = 1.74–2.00) and 1.70 (95%CI = 1.64–1.76) times greater than when observers memorized only one item, for target azimuth and elevation, respectively. These values are consistent with previous estimates of the effect of memory load on recall error [8]. These findings suggest that spatial WM depends on spatially curtailed representations, whose resolution scales with the overall memory load and the target location.

### Memory load modulates systematic errors

**Figure 2:**
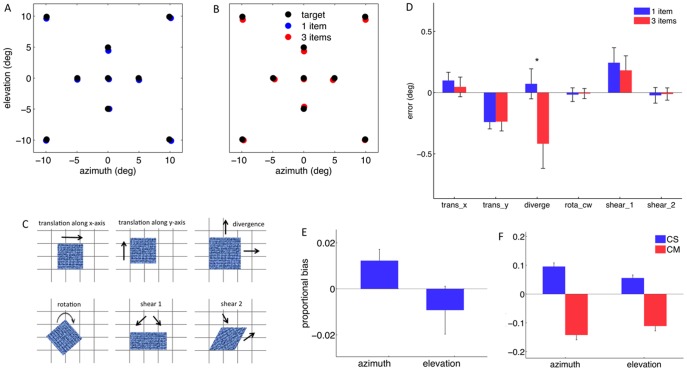
Memory load affects systematic recall error. (A) Recalled targets were systematically displaced outward and downward (in blue) relative to their location in the sample display (in black) when the memory load was one. (B) Recalled targets were displaced toward the center of the display when the memory load was three (in red). (C) The six spatial components of the systematic error are shown, including constant offsets (translation) along azimuth and elevation, and four linear tensors. (D) Memory load only affected the divergence of the error field. For the sake of convenience, the error size is expressed in degrees for the tensors as well. These values correspond to the displacement associated with each component, averaged over all target locations. (E) Proportional recall bias in center of screen (CS) coordinates (in blue) when the memory load is one. (F) Proportional recall bias in CS (in blue) and center of the memory items' configuration (CM) coordinates (in red) when the memory load is three. Target azimuth, in CS coordinates, was overestimated both when the memory load was one and three. In addition, when the memory load was three, participants underestimated both target azimuth and elevation in CM coordinates. *trans* - translation, *diverge* - divergence, *rota_cw* - clockwise rotation. *p<0.05.

It is known that recall of spatial information from working memory shows systematic distortions, which depend on both stimulus and task related factors [28]–[30]. Some have also suggested that these biases reflect the reference frames used to encode spatial data in memory [31]. We characterized the spatial structure of systematic recall errors, separately for the two levels of memory load employed. Two patterns of systematic recall errors were found. Figure 2A shows that when the memory load was one, participants overestimated the target's distance from the display center, more prominently so along azimuth than elevation. Moreover, observers recalled the target at a lower elevation than its location in the sample display warranted. However, when memory load was three, participants tended to underestimate the target's distance from the center of the screen (Figure 2B). Two-way, repeated-measures ANOVAs showed a significant interaction of target location by memory load (azimuthal - F(8,56) = 3.11, p = 0.006; elevational - F(8,56) = 3.09, p = 0.006) on the systematic error, thus confirming that the systematic recall error was modulated by memory load.

To characterize the spatial structure of recall inaccuracies and gain further insight into the nature of the load effects, we reparametrized the systematic recall error using a set of four tensors, namely the divergence, rotation and two shear components of the vector error field (Figure 2C). Paired-samples t-tests indicated that of the four tensors, only the divergence of the error field (t(7) = 3.36, p = 0.012; Figure 2D) showed a significant effect of memory load, suggesting that the effects of memory load can be characterized using a single spatial component, namely the tendency to either under or overestimate the target distance from the display center.

### Spatial memory representations are based on multiple reference frames

Next, we investigated why memory load affects spatial distortions in recall. We observed that when participants had to keep three items in memory, the reported target location was shifted toward the locations occupied by the other two memory items, suggesting that spatial distortions, when the memory load increases, arise in a reference frame centered on the memory items. Spatial distortions were thus modeled using two sets of linear regressors. The first set consisted of the target location in screen coordinates (CS), the second of the target location in the center of the memory items' configuration coordinates (CM). The target azimuth in CS coordinates was overestimated, when the memory load was both one (azimuth - t(7) = 2.48, p = 0.042; for elevation - t(7) = −0.89, p = 0.40) and three (azimuth - t(7) = 7.50, p<0.001; elevation - t(7) = 5.27, p = 0.001), however the target location in CM coordinates was underestimated (azimuth - t(7) = −8.48, p<0.001; elevation - t(7) = −6.59, p<0.001), when memory load was three (Figures 2E,F), suggesting that the change in the direction of the systematic error with memory load reflects the additive effects of two spatial representations, arising in two different reference frames.

**Figure 3:**
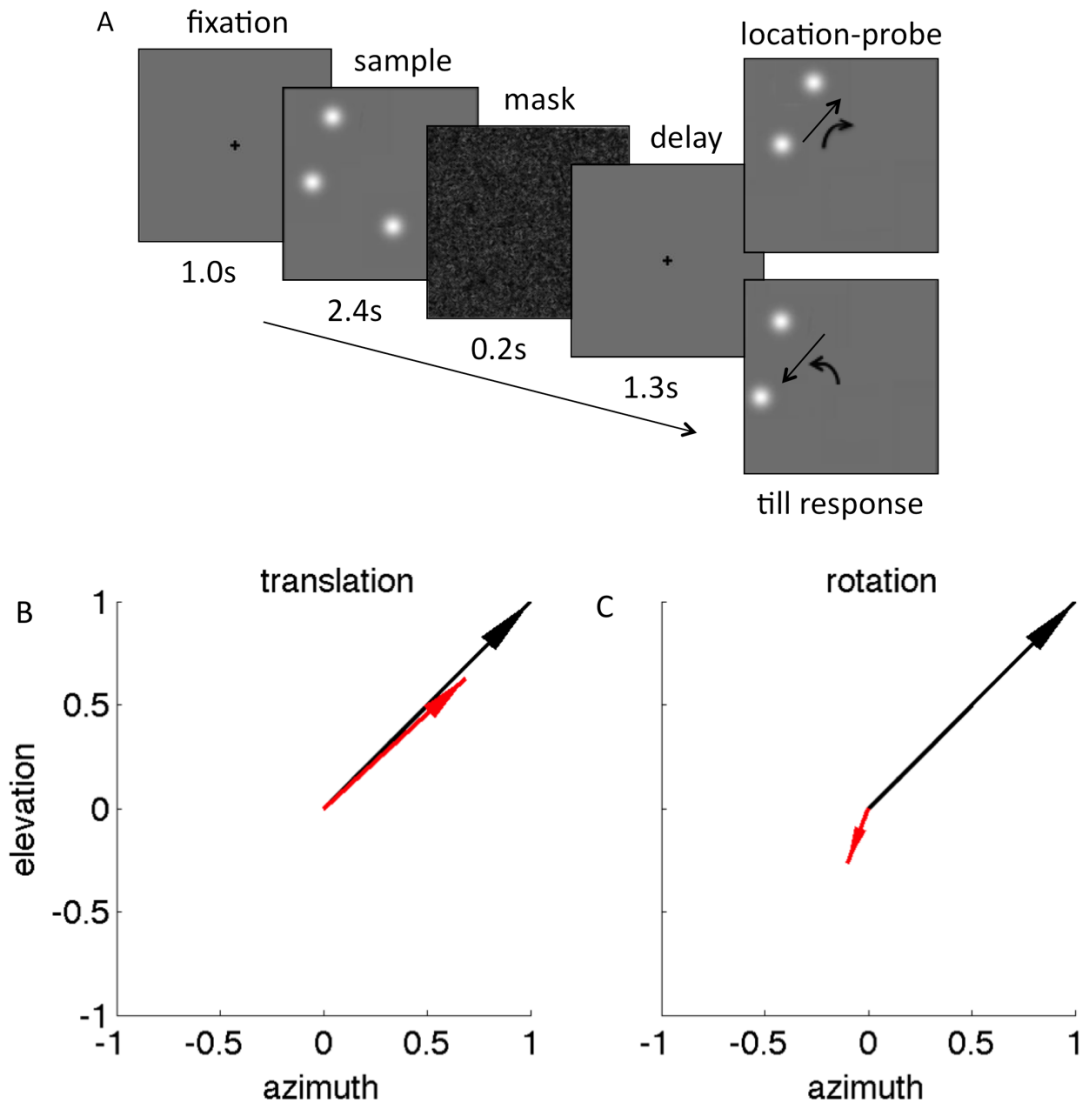
Location-probe displacement affects target recall. (A) The target was identified by displaying the position of the non-target memory items at recall. The position of the non-target items was either translated obliquely (straight arrows) or rotated around an axis through the display center (curved arrows). (B) Translation of the non-target items, whose direction and magnitude is portrayed by a black arrow of normalized length, caused the recalled target location, portrayed by the red arrow, to be displaced in the same direction, albeit by a smaller magnitude. (C) In contrast, following rotation, the recalled target location was displaced in a direction opposite the one required to preserve the distances between the memory items. For illustrative purposes, the displacement of the non-target memory items is represented by the black line and the average displacement of the target items by the red line.

The spatial configuration effects we observed may arise either because spatial data are smeared by vWM or because participants hedge their bets at recall, reporting an intermediate location, when they are not fully confident about which of the memory items is the recall target, and not because the location of the items held in memory is encoded in a reference frame centered on CM. To examine this possibility, in experiment 2, the target was identified by presenting the non-target memory items at recall, but the non-target items were either translated away from the position they had occupied in the sample display 0.6° up and to the right or 0.6° down and to the left (Figure 3A), or rotated around an orthogonal axis passing through the display center, which resulted in a mean displacement of the memory items of 0.6° (also Figure 3A). These trials were randomly intermixed within blocks in which the remaining 80% of the trials were from experiment 3. As shown in Figure 3B, the recalled target location was shifted on average by 0.4° in the same direction along which the memory items had been translated (azimuth - t(3) = 10.06, p = 0.002; elevation - t(3) = 6.10, p = 0.009), suggesting that participants memorized and reported the target location relative to the other two items' position. One possibility is that translating the non-target items shifts the origin of the reference frame, namely the CM, used to recall the target location. The other is that participants may have reported the target location, which preserves the distance of the target from the two non-target items. If the latter interpretation is correct, then displacing the non-target memory items' position by rotation at the time of recall should result in an identical rotation of the recalled target location. Participants instead recalled the target location at a position rotated by 0.11° in the direction opposite the one used to displace the non-target memory items (Figure 3C). These findings suggest that participants did not simply memorize the relative distance between the items held in memory, but rather they encoded and recalled the position of the memory items in a reference frame centered on CM. This strategy is perhaps automatic, since participants were not informed that the non-target items' location at recall may be displaced. Moreover, enquiry after completing the experiment revealed that participants had failed to notice that the location of non-target items was occasionally changed at recall.

### Further evidence for multiple reference frames based memory representations

**Figure 4:**
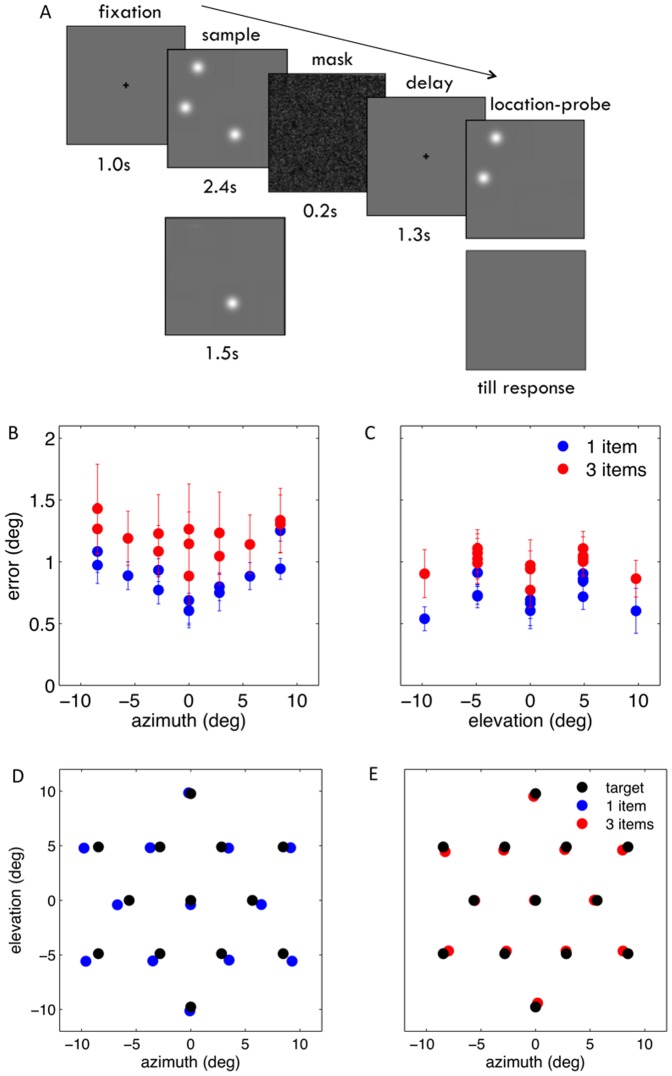
Location-probe diminishes the effects of memory load on recall. (A) Trial structure. When the memory load was three, the target to be recalled was identified by highlighting the location of the two non-target items. When the memory load was one the appearance of the cursor indicated the beginning of the recall period. (B) The error standard deviation is shown as a function of target azimuth, when the memory load is one (in blue) and three (in red), and (C) as a function of target elevation. Along azimuth, the variable error increases monotonically with target eccentricity. Along elevation, however, the variable error shows a peak at eccentricities intermediate between the display center and its boundaries. (D) Recalled targets were systematically displaced outward and downward (in blue) relative to their location in the sample display (in black) when the memory load was one, (E) while they were displaced toward the center of the display when the memory load was three (in red). (see also Figures S1A-C).

To further examine the effect of target location and configuration of the memory items on recall, we changed the display's aspect ratio in experiment 3, thus modifying the distance between display boundaries and the target. In addition, we examined the effects of providing information about the memory configuration by presenting the non-target memory items at recall. The memory items were bright discs of uniform hue. The recall display contained the two non-target items, if the sample display had contained three items, or nothing, if the sample display had contained only one item (Figure 4A). The native aspect ratio of the display (44.76°×25.84°) was used, resulting in targets being farther away from boundaries along the display azimuth than elevation. Both memory load and target location affected the variable error (See Data S1 for relevant statistics). The variable error along elevation showed a modal relation with target location, similar to the one in experiment 1, being largest for targets at locations intermediate between the display center and boundaries. However, along azimuth, the variable error increased monotonically with increasing target eccentricity, suggesting that recall error reflects the target's proximity to visual landmarks, such as the display boundaries (Figure 4B,C). The patterns of systematic errors were similar to those observed in experiment 1 (Figure 4D,E; see also Figures S1A-C; see Data S1 for relevant statistics).

We also found that the effect of memory load on recall precision was diminished - the ratio of the errors' standard deviations when the memory load was three and one, respectively, was 1.38 for azimuth, (95% CI = 1.26–1.49) and 1.36 for elevation (95% CI = 1.26–1.46). The diminished effect of memory load on precision suggests that non-target memory items, when shown at recall, may act as landmarks.

### Improved spatial recall reflects recall rather than encoding strategies

**Figure 5:**
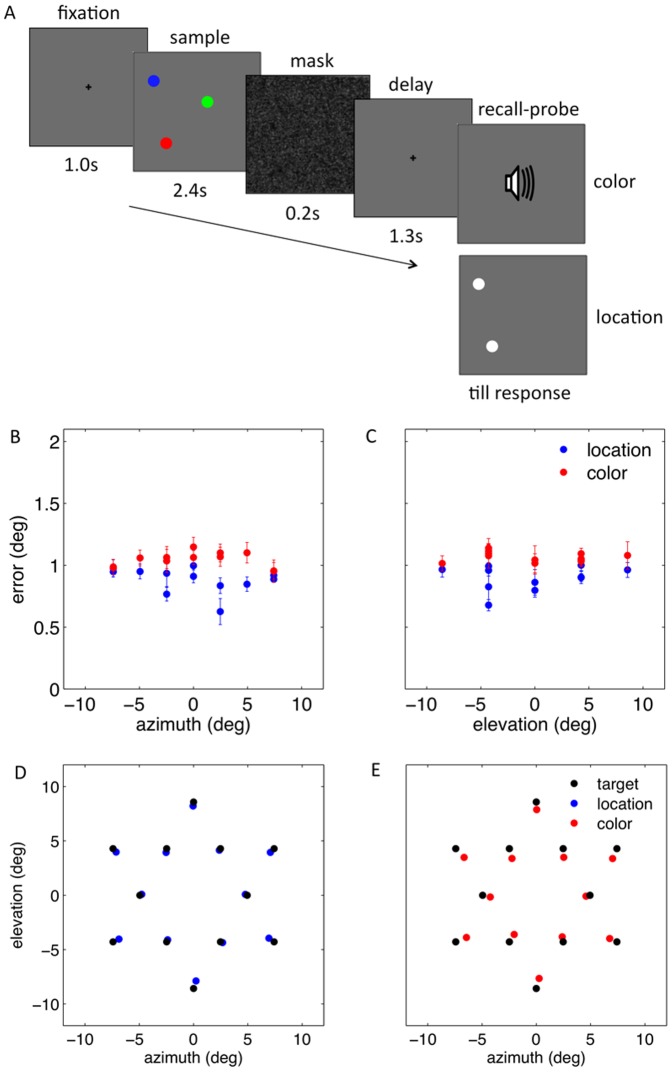
Probing procedure affects spatial recall. (A) Trial structure. The target to be recalled was identified either by presenting the non-target items (location-probe), or by voicing the target color (color-probe). The memory load was always three. (B) The error standard deviation is shown as a function of target azimuth, following location (in blue) and color-probes (in red), and (C) as a function of target elevation. Variable error was smaller for the location than the color-probe condition. This difference was largest for targets halfway between the center and the boundaries of the display. (D) Following location-probes, participants underestimated the target distance from the center of the screen less prominently than (E) following color-probes. (see also Figures S2A,B; Figures S3A-D, Figures S4A,B).

The diminished effect of memory load, when the non-target memory items were presented at recall may be confounded by differences in the display layouts and target locations used in experiments 1 and 3. In experiment 4, we compared the effects of the two recall procedures. The sample display contained three memory items and, at the time of recall, the target was identified, in separate blocks, either by its color or by presenting the non-target items. The display layout and the location of the memory items were the same in the two recall conditions (Figure 5A). When the target was identified by the non-target items' locations, the standard deviation of the recall error was greater than when the target was identified by its color, both for target azimuth (F(1,5) = 26.11, p = 0.004) and elevation (F(1,5) = 14.05, p = 0.013). Interestingly, the recall error also showed a significant interaction between recall procedure and target location along azimuth (F(11,55) = 2.04, p = 0.042), but not elevation (F(11,55) = 1.65, p = 0.109), the difference between error sizes being largest for target locations intermediate between the center and the boundaries of the display (Figures 5B,C). This observation suggests that the presence of non-target items at recall improves precision more prominently for targets farthest from landmarks. The most obvious interpretation of this trade-off is that memory items, shown at recall, act as vicarious landmarks. We also observed smaller systematic recall errors when the recall probe was the non-target items than the target color, although the patterns of errors were similar (Figures 5D,E; see also Figures S2A,B; see Data S1 for relevant statistics).

An alternative explanation for the effects of probing procedure on recall is that the demands of remembering the color of the memory items, in addition to their location, may increase the memory load when the probe is the target color and thus contribute to the differences observed between the two probing procedures. This explanation appears unlikely given that different dimensions of the visual stimulus, such as position and color, are thought to be encoded using independent memory resources [9], [32]. A perhaps more plausible hypothesis is that participants could have used different encoding strategies depending on the probe used to identify the target at recall. For example, observers may have memorized only the average location of the memory items' configuration, when the location-probe was used, since a simple computation would then yield the location of the target item at recall. In experiment 5, we controlled for these potential confounds by mixing probes in the same blocks. Since the participants could not anticipate which recall probe would be used, they had to remember both the location and color of the memory items in every trial, thus minimizing the possibility that memory load or encoding strategies could contribute to performance differences between probing procedures. We observed smaller variable and systematic errors in the location than the color-probe condition (Figures S3A-D and S4A,B; see Data S1 for relevant statistics), as in the previous experiment, suggesting that the probe effects arise at recall rather than reflecting changes in encoding strategies.

## Discussion

### Local level spatial representations determine recall precision

Recent vWM models have assumed that visual information is stored in buffers of limited resolution that may be specific for an object's feature dimensions, be its color [7], orientation or location [8], but are otherwise not tuned to particular features within a dimension, for example red vs. blue in the case of color. Accordingly, the ability to recall precisely the color of a target, its orientation or location should not further depend on its specific color, orientation or location. However, we found that the precision of spatial recall was significantly modulated by target location and, even more importantly, that memory load scaled multiplicatively the magnitude of the recall error at each of the target locations. Because target location and memory load interact, rather than exert independent, additive effects on recall error, we conclude that the precision of spatial recall depends on the resolution of spatially tuned mechanisms, instead of mechanisms that generalize over space.

An alternative explanation that could be put forth to explain the multiplicative interaction between the memory load and target location is that these effects arise at the perceptual level due to increase in perceptual noise with increasing number of items. This seems unlikely, as our participants were encouraged to foveate each individual item during the encoding phase and the sample display was visible long enough to render that possible. Furthermore, if the memory load effects observed are perceptual in nature, decreasing the perceptual noise by increasing the duration of the display during encoding should decrease the memory load effects. However, previous studies have shown that memory load effects are largely independent of the display duration [6], [7]. Also, increasing the delay interval between encoding and recall should have no effect on the recall error, if these effects are perceptual in nature. Contrary to this prediction, Sheth and Shimojo (2001) showed that the recall error of a target location towards a visually salient landmark systematically increases with increasing delay interval [31]. Considering all these, a perceptual explanation of the effects observed in our study seems unlikely.

The effects of target location suggest that proximity to landmarks, whether the boundaries or the center of the display, improved recall precision. In fact, when the vertical boundaries of the display were displaced outwards, thus increasing their distance from the most eccentric targets, these targets were recalled less precisely than targets at intermediate positions. Finally, an improvement in recall precision was observed particularly for targets that were farthest from both center and the boundaries of the display, when non-target memory items were presented at recall suggesting that they acted as vicarious landmarks. Visual landmarks have been found to improve the localization of nearby visual targets under conditions in which perceptual rather than memory constraints were examined [33], suggesting perceptual and memory representations, which guide target localization, are largely shared. This inference is in keeping with recent functional imaging data concerning the neurophysiological underpinnings of visual short term memories in humans, which demonstrated that distributed, voxel-wise patterns of BOLD signals in early visual cortical regions, recorded while participants maintained visual data in memory, convey information about memorized stimuli [18]–[22]. Our behavioral data, in contrast to current vWM models, are consistent with this neurophysiological evidence because they indicate that vWM utilizes spatially tuned processes similar to those used during the analysis of the sensory input in visual cortex.

An issue of significant theoretical interest is why the resolution of local representations of visual data should be liable to the effects of memory load. Specific, though speculative, proposals have been put forth by others, including the suggestion that the spatial resolution may be limited by within receptive field interactions among closely spaced memory items [34], [35] or population level normalization of neural activity in cortex [36].

### Global spatial representations anchor local data to stimulus configuration

Local visual representations seem to account for the effects of target location on recall precision. However, we also found that interactions among the elements that make a visual scene affect recall accuracy. These effects do not seem to be spatially limited, but rather increase with their distance from the recalled target [29]. When the observers had to remember only one item, the recalled target location was displaced eccentrically, that is away from the center of the display. However, when three items had to be remembered, the recalled target location was displaced toward, rather than away from center of the display. The direction change of the recall bias with memory load was found to reflect two linearly independent distortions. The first was a hypermetric bias in display coordinates, which solely determined the nature of the systematic error when one item was memorized, and a hypometric bias toward the center of the memory items' configuration, which dominated the systematic error when three items were memorized. However, the persistence of the hypermetric bias in screen coordinates when three items were held in memory, suggests separate causes for the hypermetric and hypometric biases. Hence, we suggest that spatial memory depends on representations which employ separate reference frames, one in display coordinates and the other relative to the center of the set of memorized elements. Other interpretations of the hypometric bias are not particularly plausible. For example, it is possible that participants may have occasionally reported the location of a non-target memory item, giving the impression that the recalled location of the target was displaced, when averaged over trials, toward the center of the memory items' configuration. However, we excluded from the analysis trials in which the recalled location was closer to one of the non-target memory items than the target. Moreover, when the recall display contained two of the memory items, the target was still recalled toward the center of the memory items' configuration, albeit by a diminished amount, indicating that when the proportion of binding errors was minimized, analytically or by the probe procedure used to identify the target, the hypometric bias in memory coordinates persisted. However, the most conclusive evidence was provided by the finding that translating the non-target items at recall caused a parallel translation of the recalled target location by a similar amount, a finding that simply cannot be explained by participants confounding target with non-target items. A second hypothesis one may entertain, is that the location of the memory items is coded in relative terms, namely that observers keep track of the distance between items in memory rather than their location in relation to some other reference. If this were the case, then any rigid displacement of the non-target memory items should result in displacement of the recalled target to preserve the overall configuration. However, when we displaced the non-target memory items at recall, by rotating their position around an axis passing through the center of the display and orthogonal to the image plane, the recalled target location was not displaced in the direction that would have preserved the distance between the target and non-target items, but rather in the opposite direction. This finding fundamentally undermines the view that observers were memorizing the distance between the target and non-target memory items, and supports instead the interpretation that the target position is coded and recalled relative to a common reference frame, namely the center of the memory items' configuration. Thirdly, it has been suggested that configurational effects on recall may be best understood within a Bayesian framework [24], [37]. Accordingly, the configuration of the memory array is used to generate a prior for the distribution of the possible target positions, which, combined with noisy estimates of the actual target position, leads to target recalls biased toward the average position of the memory items. This proposal would predict an increase in centripetal bias whenever information about the memory configuration is provided. However, we found that presenting the non-target items at recall diminishes the centripetal bias. Single unit data in higher order parietal areas of behaving primates have indicated that in a match to sample task, visually evoked responses to previously seen dot stimuli are largely invariant to parallel displacements of the stimulus over the retina, suggesting a stimulus centered coding of complex spatial configurations [26], [27], such as those that may underpin the behavioral effects summarized above. The maintenance of the configuration of memory arrays in higher order regions could also account for the finding that the complexity of the memory array scales the amplitude of delay period activity in parietal and frontal regions during vWM tasks [13]–[17].

In conclusion, both behavioral and neurophysiological data are consistent with the idea that there are at least two representations of spatial data in vWM - one where fine spatial details are maintained by spatially curtailed mechanisms in early visual cortex, the other where summaries of the global configuration are maintained by mechanisms that generalize over space in higher cortical areas.

### Do configurational effects arise only at encoding or also at recall?

Configurational effects in vWM are assumed to arise at the encoding stage as they are believed to reflect the consequences of perceptual grouping and gestalt effects [37], [36]. However, when non-target memory items are shown at recall, there is an improvement in accuracy and a diminished centripetal bias. Moreover, when non-target memory items were translated at recall, the recalled target location was also displaced in parallel direction. These findings indicate that configurational effects do not arise only at encoding but also at recall. One may therefore speculate that the same spatial updating mechanisms that characterize the positional invariances of parietal neurons mentioned above also characterize recall processes in human participants [26], [27].

## Experimental Procedures

### Participants

All experimental protocols were approved by the Research Ethics Committee at Bangor University. Participants gave written informed consent prior to commencing experimental procedures and were remunerated for their participation. All participants were adults with no known neurological disorder and were not taking psychotropic medications at the time of testing. Participants had normal or corrected-to-normal visual acuity and color vision.

### Stimuli and experimental procedures

#### Experiment 1

Eight participants (three females) with a mean age of 29.5 years (s.d. = 7.3 years) took part in the color-cue experiment. The experiment was controlled by a custom coded script in Matlab running on an Apple iMac 10. The visual display was controlled using a set of freely available procedures [38]–[40]. The display was an LCD monitor (NEC LCD3210). The monitor subtended 25.84°×25.84° of visual angle. Participants' head position was restrained by a chin rest, which ensured that a constant distance of 85.0 cm from the display was kept during the experiment.

Participants were instructed to fixate a central cross at the beginning of each trial and during the inter-trial interval, but were encouraged to move their eyes to examine the items of the sample array and the recall display. Participants completed initially a practice block of fifty trials to gain familiarity with the task. Each participant was subsequently tested over separate sessions, run over four consecutive days. Each session consisted of four hundred and fifty trials divided into three blocks of one hundred and fifty trials each. The memory load varied over two levels: participants remembered the location of either one or three discs. The order of trials containing either one or three memory items was randomized within blocks.

The sample display contained either one or three red, green, or blue discs displayed on a grey background. The discs occupied nine possible positions, including the center of the display and the corners of two concentric, imaginary squares tilted 45° relative to each other. The items' locations were jittered on each trial, by adding independent, identically distributed two-dimensional Gaussian noise (σ = 0.7°). The recall at each target location was probed in eighty-eight trials, when the display contained a single memory item, and in one hundred and twelve trials, when it contained three memory items. The set of display configurations containing three memory items was obtained by exhaustive combination of nine target locations and fifty-six permutations of the two non-target items.

Each trial started with a 1.0 s long presentation of a fixation cross at the center of the display. This was followed by sample array containing either one (displayed for 1.5 s) or three discs (displayed for 2.4 s), which allowed adequate time for an exhaustive inspection of each item. This was followed by a 0.2 s pattern mask containing a pseudorandom luminance pattern of bright and dark pixels. A 1.3 s long blank screen immediately followed. The target, whose location the participants were asked to replicate, was probed by an electronically recorded voice, which indicated its color. The participants reported the target location by moving a cross-hair cursor to the memorized location via a hand held mouse (Figure 1A). Participants were instructed to be as accurate as possible, without any time limitations. To ensure that participants memorized the color also when a single disc was presented, in 10% of these trials, the instructed and the target color differed; participants were asked to place the cursor at the edge of the display in trials in which target and probed color differed.

#### Experiment 2

Four participants (one female), with a mean age of 34.2 years (s.d. = 8.4 years), were tested in the recall probe translation/rotation experiment. The stimuli, apparatus and procedures were identical to those used in location probe experiment, except that it only included trials containing three memory items. Moreover, in the recall display, the position of the two memory items was either translated, down and to the left or up and to the right, or rotated clockwise or anticlockwise (Figure 3A). The displacement was 0.6° of visual angle for translation and 5° around the line of sight for rotations - resulting in mean displacement of memory items in the image plane of 0.6°. Participants were not told that the location of the non-target memory items could change at recall. These trials were mixed within blocks of experiment 3. There were eight hundred and fifty-eight trials each for translation and rotation conditions. For each participant, all possible combinations of target and non-target locations were tested an equal number of times – each location being tested sixty-six times for both the translation and rotation conditions.

#### Experiment 3

Four participants (one female) with a mean age of 34.2 years (s.d. = 8.4 years) were recruited. Procedures were identical to those used in experiment 1, except for the outline of the display, the location of the stimuli and the report procedure.

The stimuli were presented on the LCD screen using its native rectangular aspect ratio. The screen subtended 44.8°×25.8° of visual angle and was placed at a distance of 85.0 cm from the participant. Visual stimuli appeared at one of thirteen possible locations, which included the center of the display and the corners of two concentric hexagons tilted 90° relative to each other. The memory items were bright discs displayed on a grey background. The items' locations were jittered by 2D, independent identically distributed Gaussian noise (σ = 0.7°).

When three memory items were presented in the sample display, two of the memory items were shown again in the recall display. The participants were instructed to report the location of the remaining target. When the sample display contained a single memory item, the recall display included only the cursor cross-hair, which was used to indicate the target location (Figure 4A). Each participant was tested in fourteen, consecutive, daily sessions. In each session, participants completed five hundred and seven trials over three blocks comprising hundred and twenty-seven trials each and one comprising hundred and twenty-six trials. For each participant, all possible combinations of target and non-target locations were tested an equal number of times - each location being tested eighty-four times for the one memory item condition and four hundred and sixty-two times for the three memory items' condition.

#### Experiment 4

Six participants (five females) with a mean age of 20.3 years (s.d. = 1.6 years) took part in this experiment comparing the effects of location and color probes on recall. The stimuli, apparatus and procedures were identical to those used in the previous experiments, except for the memory locations, the recall procedure, and the fact that sample display always contained three stimuli. The stimuli appeared at twelve possible locations as described in experiment 3, except for the center one. In half the trials, the recall probe was the color of the target, in the other half, the locations of the non-target items (Figure 5A). The color and location probes were used in separate blocks. Each participant was tested over four, consecutive daily sessions. Each session consisted of three alternating color and location probes blocks, whose order was counterbalanced across participants. Each block contained hundred and forty-three trials each. All possible combinations of target and non-target locations were tested an equal number of times - each location being tested sixty-six times in both probe conditions.

#### Experiment 5

Ten participants (six females), with a mean age of 29.1 years (s.d. = 6.19 years), were recruited to examine the effects of mixing the two probe conditions within blocks. The stimuli, apparatus and procedures were similar to those used in experiment 4, except for the location of memory items, and the use of both color and location probes within the same block. The stimuli appeared at thirteen possible locations, including the center of the display and the corners of three concentric squares tilted 45^0^ relative to each other. The color and location probe trials were randomly intermixed, ensuring that participants memorized both the color and location of the stimuli in each trial. Each participant was tested over four daily sessions, each session consisting of three blocks of hundred and forty-three trials. All possible combinations of target and non-target locations were tested an equal number of times - each location being tested sixty-six times for both probe conditions.

### Data analysis

The aim of the analysis was to quantify the systematic and variable components of the errors made when recalling the target location. The systematic recall error was the difference between recalled and actual target location, which was consistently repeated over trials. Operationally, it was defined as the average difference between recalled and actual target location, as estimated by linear regression models (see below). The variable error was the error component, which varied from trial to trial. It was defined as the root square of the mean squared difference between recalled and actual target location, after removing any systematic difference. The reciprocal values of these two error components are referred to as *accuracy* and *precision*, respectively. The following paragraphs detail the specific procedures we used to estimate the systematic and variable errors.

#### Systematic error (recall accuracy)

The systematic error was assessed both over all target locations simultaneously, as well as separately for each canonical target location. The aim was to examine the systematic errors as a function of the memory load, and the effect of memory items' spatial configuration on the recalled target location. Prior to the analysis, trials were removed in which the reported location was closer to a non-target memory item than the target. These trials never amounted to more than 2% of the trials in all of the participants and experiments.

The following linear model was used to estimate the relation between target location and recalled location:
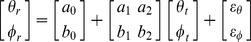
where *θ_r_* and *φ_r_* are the reported azimuth and elevation, *θ_t_* and *φ_t_* the target's azimuth and elevation, while ε_θ_ and ε_φ_ are the variable errors, assumed to be drawn from zero mean, normal distributions with degrees of freedom equal to the number of trials minus three. The parameters of the model were estimated using a least square procedure. The above model was applied to data obtained at each target location, using only trials in which the target appeared, after its location had been jittered, within 2.5° of its canonical location. The recalled locations, for each canonical target position, were estimated by substituting the coordinates of the canonical target location in the model above.

To further examine the global effects of memory load on target recall, the model was used to analyze data, which included trials from all target locations, separately for each level of the memory load. The estimated parameters were transformed into tensors of the systematic error field. This set included the divergence, curl and two shear components of the systematic error vector field. These tensors captured respectively the tendency to overestimate or underestimate 1) the target distance from the display center, 2) the orientation of the target relative to the center of the display, 3) the target distance from the display center unequally along the horizontal and vertical axes, and 4) the target distance from the display center unequally along the two main oblique axes of the display (Figure 2c). The tensors were computed using the following formulas:
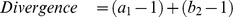





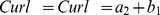





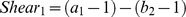





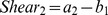



When three items were memorized, the systematic error was also estimated using a linear model, whose regressors included the target location in screen coordinates as well as in center of the memory items' configuration (CM) coordinates. The CM coordinates of the target (*θ ^cm^*, ***φ^cm^***) were computed from its (*θ^ s^*, ***φ^s^***) and the non-target memory items screen coordinates, using the following expression:
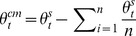





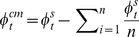
where 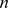 is the number of memory items. The linear model thus contained four regressors, in addition to the constant terms: 
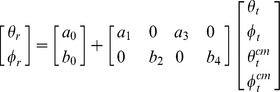



The parameters which modelled the crossed effects of the target azimuth and elevation, in screen and CM coordinates, on the recalled target elevation and azimuth respectively, were set to zero, since a preliminary analysis indicated that their group level contribution to the linear fits was neglicable.

#### Effects on target recall of translating and rotating the position of non-target items

In experiment 2 we either translated or rotated the position of the non-target items at recall. The effect of these positional changes on target recall was estimates using a linear regression procedure.

Translation of the non-target items took place always along the main oblique axis, either down and to the left or up and to the right. Therefore the displacement of the non-target items along azimuth and elevation had the same magnitude *T*. The effect on the recall of the target azimuth and elevation could then be estimated using the following model:
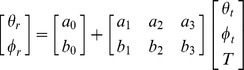



The horizontal and vertical components of the red line shown in Figure 3B are the group averaged values of *a_3_* and *b_3_* respectively.

Rotation of the non-target items position took place around an orthogonal axis through the screen center. We estimated the effects of rotation on target recall along the direction, which would have resulted in a rigid rotation of the target and non-target memory items. The target displacement, which would have resulted in a rigid rotation of the array, was computed on each trial using the following equation:
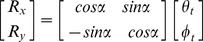
where α is the angle of rotation around the orthogonal axis and *R_x_* and *R_y_* are the displacements of the target azimuth and elevation, respectively, required to maintain the rigid configuration of the memory array. The effect of non-target items rotation on target recall was then computed using the following model.
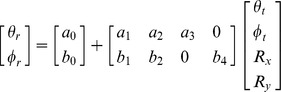



The horizontal and vertical components of the red line shown in Figure 3C are the group averaged values of *a_3_* and *b*_4_ respectively.

#### Recall variable error (recall precision)

The azimuthal and elevational components of the variable error were estimated at each target location. For each target location, the systematic recall error was computed using a separate linear model, as described above. The residuals *ε_1,…,n_* of the linear model, over *n* trials, were rank ordered and their cumulative probability score *p_r_* was computed from their rank, *r*, as follows: 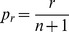



The cumulative probabilities were transformed into *z* scores using the probit function: 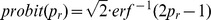



The computation of the linear model and the residuals was then repeated, the second time after trials, whose *z* scores lay outside the interval (−1.8, 1.8), had been excluded. However, the *z* scores were not recomputed.

The standard deviation of the error was estimated by fitting the following model:
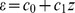
where ε is the set of variable errors and *c_1_* the estimated standard deviation σ of the variable errors, that is:
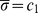



This procedure provides an estimate of the error standard deviation that is robust in the face of outliers due, for example, to accidental mouse clicks or guesses.

#### Assessing the effects of memory load on variable error

A central issue in this work is the nature of the relation between the effects of target location and memory load on recall variable error. First, we examined whether target location and memory load exert independent, additive effects on the recall error variance, as one would predict if the effects of target location and those of memory load arise at different processing stages. If target location and memory load exert independent effects then the recall error variance should simply be the sum of the variances due to each, and show no interaction between these two factors.

Having found that the target location and memory load interact (see Results), we then examined the nature of this interaction. We assumed that the recall error at each location varied multiplicatively with the memory load. In other words, the ratio between the variances of the recall error, when the memory load was three and one respectively, was constant across target locations:
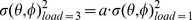



We compared this multiplicative model to a model, which assumed that the variance of the recall error when the memory load is three, is the sum of two components: 1) the error made when the memory load is one, whose variance varies with target location, and 2) an error of constant variance, which does not change with target location:
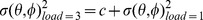



The parameters of the two models, namely *a* and *c*, were estimated separately for each observer using a maximum likelihood procedure. The log-likelihood, Λ, for the multiplicative model was computed by integrating the product of two posterior chi-squared distributions of the measured error variances, at each target location, and by summing their logarithms over locations:
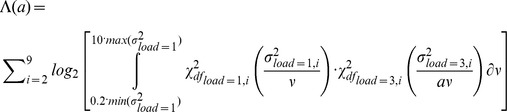



The log-likelihood for the additive model was computed similarly:
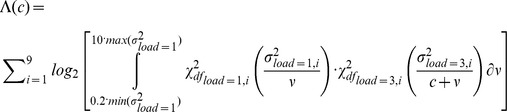



The limits of integration were set between two values corresponding to a fifth of the smallest and ten times the largest variance, when the memory load was one. A simplex algorithm was used to search for the values of the parameters *a* and *c*, which maximized the respective likelihood functions. The two maximum log-likelihoods were then compared to establish which of the two models provided a better fit to the data.

## Supporting Information

Figure S1 **Related to** **Figure 4** **; Memory load effects on systematic error.** (A) Memory load only affected the divergence of the error field. (B) Proportional recall bias in center of screen (CS) coordinates (in blue) when the memory load is one. (C) Proportional recall bias in CS (in blue) and center of the memory items' configuration (CM) coordinates (in red) when the memory load is three. Target azimuth and elevation, in CS coordinates, was overestimated both when the memory load was one and three. In addition, when the memory load was three, participants underestimated both target azimuth and elevation in CM coordinates. *trans* - translation, *diverge* - divergence, *rota_cw* - clockwise rotation. **p<0.01.
(TIF)

Figure S2 **Related to** **Figure 5** **; Probing procedure effects on the systematic error.** (A) Probing procedure only affected the divergence of the error field. (B) Proportional systematic bias in screen (CS) coordinates along azimuth and elevation, and center of memory items' configuration (CM) coordinates along azimuth and elevation following location (in blue) and color-probes (in red). Target azimuth, in CS coordinates, was overestimated whether the probe was location or color. Along the azimuth, significantly smaller displacements of the recalled target locations towards the CM were observed following location than color-probes. *trans* - translation, *diverge* - divergence, *rota_cw* - clockwise rotation, *azim* - azimuth, *elev* - elevation. *p<0.05.
(TIF)

Figure S3 **Related to** **Figure 5** **; Probing procedure effects on spatial recall in mixed-probe blocks.** (A) The error standard deviation is shown as a function of target azimuth, following location (in blue) and color-probes (in red), and (B) as a function of target elevation. The variable error was smaller following location than color-probes. This difference was largest for targets between the center and the boundaries of the display. (C) Following location-probes, participants underestimated the target distance from the center of the screen less prominently than (D) following color-probes.
(TIF)

Figure S4 **Related to** **Figure 5** **; Probing procedure effects on the systematic error in mixed-probe blocks.** (A) Probing procedure only affected the divergence of the error field. (B) The proportional systematic bias in screen (CS) and center of memory items' configuration (CM) coordinates, along azimuth and elevation, is shown following location (in blue) and color-probes (in red). Along azimuth as well as elevation, significantly smaller displacements of the recalled target locations towards the CM were observed following the location than the color-probe. *trans* - translation, *diverge* - divergence, *rota_cw* - clockwise rotation, *azim* - azimuth, *elev* - elevation. **p<0.01, ***p<0.001.
(TIF)

Data S1 **Related to Results, Statistical analysis of experiments 2, 4 and 5.**
(DOCX)
