# Supplementary figures and images for: Fine-Grained, Local Maps and Coarse, Global Representations Support Human Spatial Working Memory
Source: PLoS One. 2014 Sep 26;9(9):e107969. doi: 10.1371/journal.pone.0107969 (PMC4178058; doi:10.1371/journal.pone.0107969)

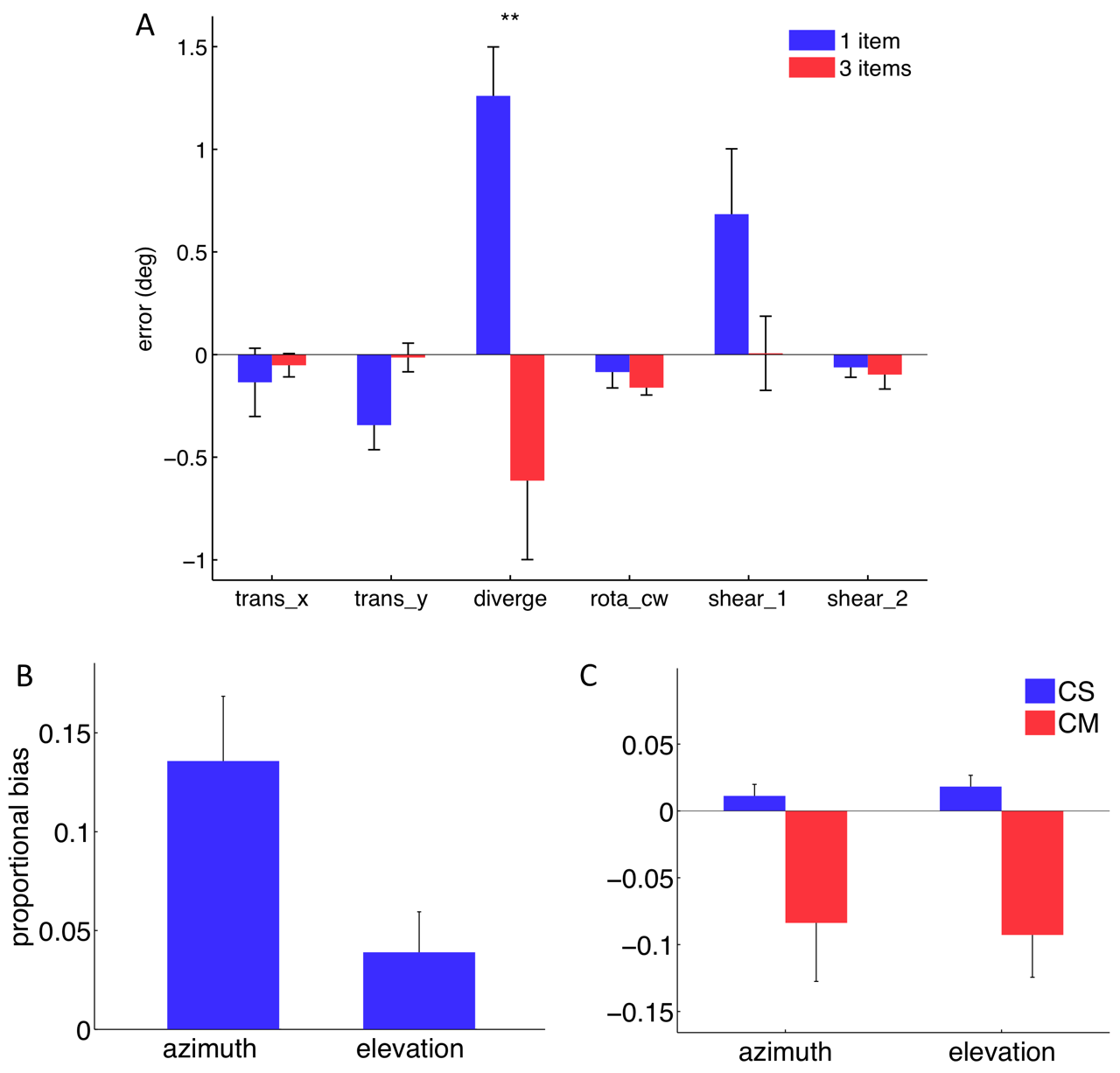

Supplement: Figure S1 — Related to Figure 4 ; Memory load effects on systematic error. (A) Memory load only affected the divergence of the error field. (B) Proportional recall bias in center of screen (CS) coordinates (in blue) when the memory load is one. (C) Proportional recall bias in CS (in blue) and center of the memory items' configuration (CM) coordinates (in red) when the memory load is three. Target azimuth and elevation, in CS coordinates, was overestimated both when the memory load was one and three. In addition, when the memory load was three, participants underestimated both target azimuth and elevation in CM coordinates. trans - translation, diverge - divergence, rota_cw - clockwise rotation. **p<0.01. (TIF) [file pone.0107969.s001.tif]

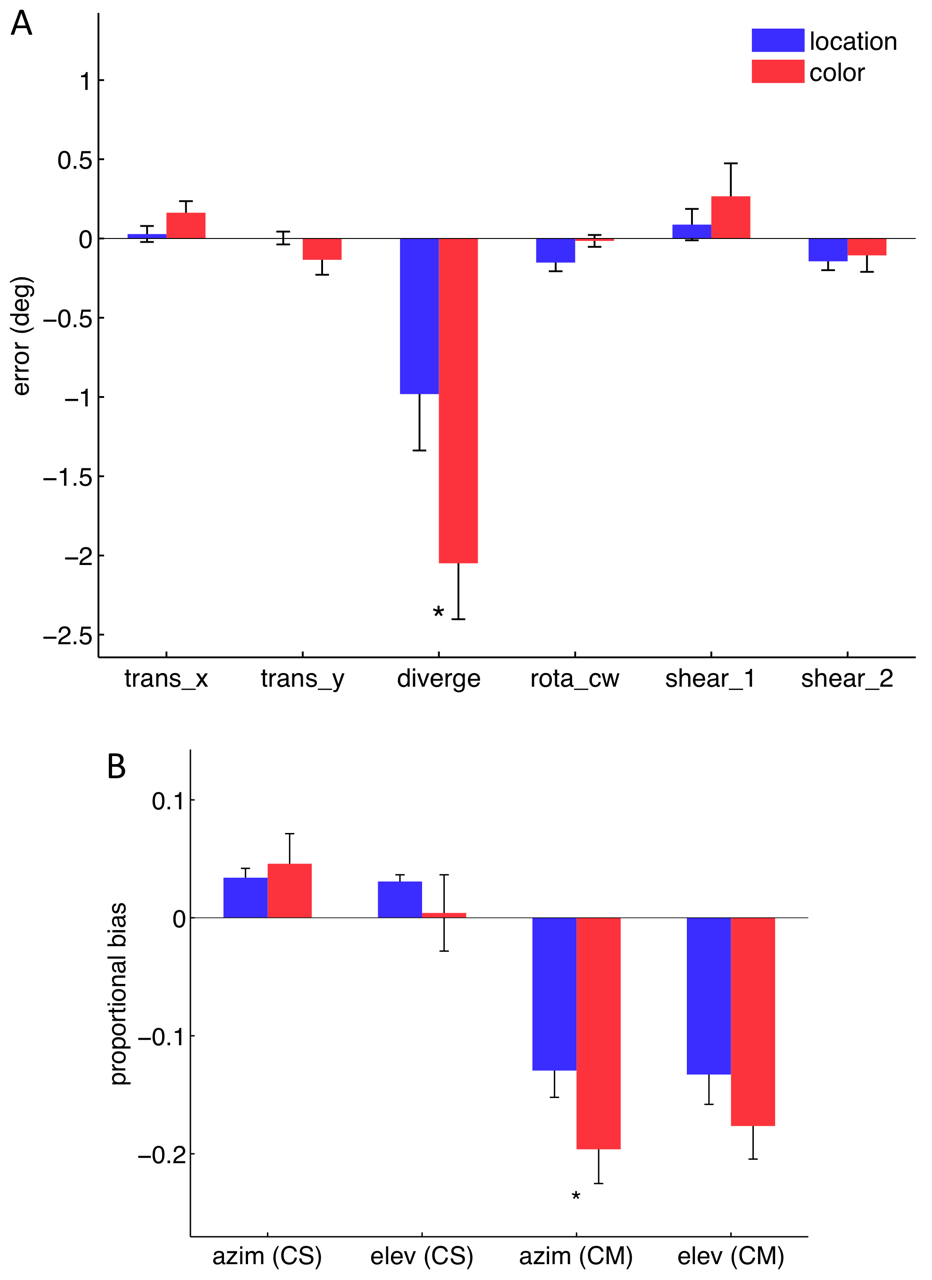

Supplement: Figure S2 — Related to Figure 5 ; Probing procedure effects on the systematic error. (A) Probing procedure only affected the divergence of the error field. (B) Proportional systematic bias in screen (CS) coordinates along azimuth and elevation, and center of memory items' configuration (CM) coordinates along azimuth and elevation following location (in blue) and color-probes (in red). Target azimuth, in CS coordinates, was overestimated whether the probe was location or color. Along the azimuth, significantly smaller displacements of the recalled target locations towards the CM were observed following location than color-probes. trans - translation, diverge - divergence, rota_cw - clockwise rotation, azim - azimuth, elev - elevation. *p<0.05. (TIF) [file pone.0107969.s002.tif]

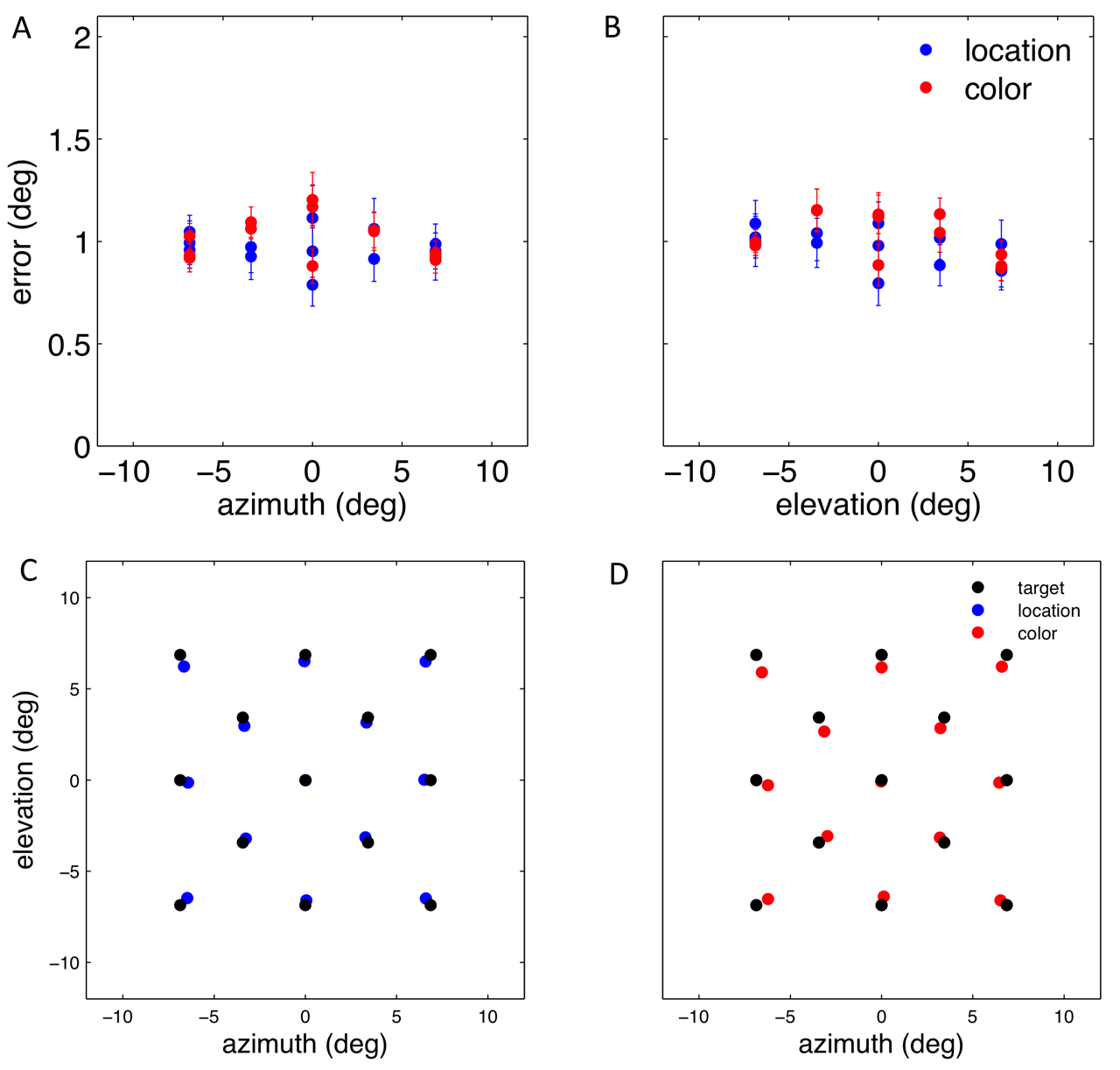

Supplement: Figure S3 — Related to Figure 5 ; Probing procedure effects on spatial recall in mixed-probe blocks. (A) The error standard deviation is shown as a function of target azimuth, following location (in blue) and color-probes (in red), and (B) as a function of target elevation. The variable error was smaller following location than color-probes. This difference was largest for targets between the center and the boundaries of the display. (C) Following location-probes, participants underestimated the target distance from the center of the screen less prominently than (D) following color-probes. (TIF) [file pone.0107969.s003.tif]

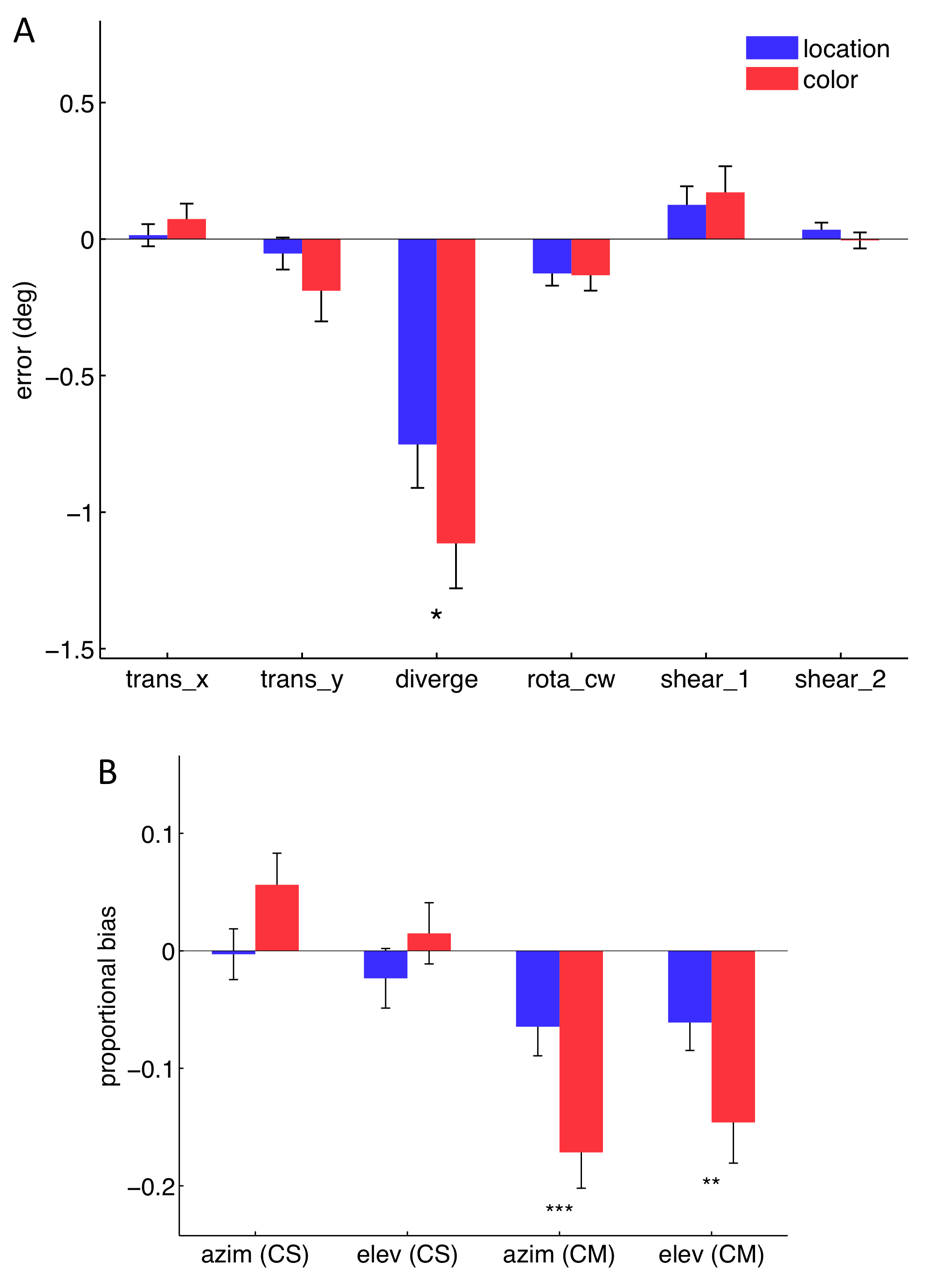

Supplement: Figure S4 — Related to Figure 5 ; Probing procedure effects on the systematic error in mixed-probe blocks. (A) Probing procedure only affected the divergence of the error field. (B) The proportional systematic bias in screen (CS) and center of memory items' configuration (CM) coordinates, along azimuth and elevation, is shown following location (in blue) and color-probes (in red). Along azimuth as well as elevation, significantly smaller displacements of the recalled target locations towards the CM were observed following the location than the color-probe. trans - translation, diverge - divergence, rota_cw - clockwise rotation, azim - azimuth, elev - elevation. **p<0.01, ***p<0.001. (TIF) [file pone.0107969.s004.tif]
