# Supplementary material for: Fine-Grained, Local Maps and Coarse, Global Representations Support Human Spatial Working Memory
Source: PLoS One. 2014 Sep 26;9(9):e107969. doi: 10.1371/journal.pone.0107969 (PMC4178058; doi:10.1371/journal.pone.0107969)
Supplement: Data S1 — Related to Results, Statistical analysis of experiments 2, 4 and 5. (DOCX) [file pone.0107969.s005.docx]

**SUPPLEMENTAL DATA S1**

**(Related to Results)**

**Experiment 2**

Two-way, repeated-measures ANOVAs, revealed a significant effect of target location (azimuthal - F(12,36) = 6.73, p < 0.001; elevation – F(12,36) = 3.93, p = 0.001), and a significant interaction between target location and memory load (azimuthal - F(12,36) = 2.89, p = 0.007; elevational - F(12,36) = 2.02, p = 0.052) on variable error. However, no main effect of memory load was found (azimuthal - F(1,3) = 3.83, p = 0.145; elevational - F(1,3) = 7.05, p = 0.077; Figures 4B,C).

Two-way, repeated-measures ANOVAs showed a significant effect of target location (azimuthal - F(12,36) = 2.90, p = 0.007; elevational - F(12.36) = 0.18, p = 0.999), but no significant effect of memory load (azimuthal - F(1,3) = 0.31, p = 0.615; elevational - F(1,3) = 4.34, p = 0.129) on systematic error. A significant interaction between target location and memory load (azimuthal - F(12,36) = 23.10, p < 0.001; elevational - F(12,36) = 17.40, p < 0.001; Figures 4D,E) was observed.

Reparametrizing the linear component of error in terms of tensors showed a significant memory load effect on the error divergence, namely the tendency to overestimate/underestimate the target distance from the display center (t(3) = 7.78, p = 0.004), as in experiment 1 (Figure S1A). The systematic recall errors in CS and CM coordinates were broadly similar to those found in experiment 1 (Figures S1B,C). One sample t-tests showed that the target azimuth, but not elevation, was overestimated in CS coordinates (azimuth - t(3) = 4.14, p = 0.026; elevation - t(3) = 1.91, p = 0.153), when the memory load was one. When the memory load was three, one sample t-tests showed no significant systematic error in CS coordinates (azimuth - t(3) = 1.30, p = 0.286; elevation - t(3) = 2.16, p = 0.119), and a marginally significant underestimation in CM coordinates along elevation only (azimuth - t(3) = -1.91, p = 0.152; elevation - t(3) = -2.93, p = 0.061).

**Experiment 4**

The systematic recall errors were diminished when a location probe was used compared to a color probe. There was no significant main effect of probing procedure (azimuth - F(1,5) = 2.63, p = 0.166; elevation - F(1,5) = 3.56, p = 0.118). There was a significant effect of target location (azimuth - F(11,55) = 11.19, p < 0.001; elevation - F(11,55) = 17.25, p < 0.001), as well as a probing procedure by target location interaction (azimuth - F(11,55) = 3.93, p < 0.001; elevation - F(11,55) = 9.13, p < 0.001; Figures 5D,E). Paired-samples t-tests showed that probing procedure significantly affected only the divergence of the error fields (t(5) = 3.08, p = 0.027), the distance of the target from the display center being underestimated more following the color than the location probe (Figure S2A).

A two-way, repeated-measures ANOVAs showed that the recall error was significantly affected by probing procedure (azimuth - F(1,5) = 10.10, p = 0.025; elevation - F(1,5) = 10.40, p = 0.023) and reference frame (azimuth - F(1,5) = 33.91, p = 0.002; elevation - F(1,5) = 26.77, p = 0.004) (Figure S2B). The probing procedure by reference frame interaction was not significant (azimuth - F(1,5) = 5.58, p = 0.065; elevation - F(1,5) = 0.09, p = 0.777).

**Experiment 5**

Two-way, repeated-measures ANOVAs showed no significant effect of the probing

procedure (azimuth - F(1,9) = 0.23, p = .643; elevation - F(1,9) = 0.30, p = 0.598), but a significant effect of target location (azimuth - F(12,108) = 3.58, p < 0.001; elevation - F(12,108) = 6.46, p < 0.001), and probing procedure by target location interaction (azimuth - F(12,108) = 2.66, p = 0.004; elevation - F(12,108) = 2.06, p = 0.025) on the recall error standard deviation (Figures S3A,B). The systematic recall errors were smaller following the location probe (Figures S3C,D). Two-way, repeated-measures ANOVA showed no significant effect of probing procedure (azimuth - F(1,9) = 4.19, p = 0.071; elevation - F(1,9) = 3.92, p = 0.079), but a significant effect of target location (azimuth - F(12,108) = 14.26, p < 0.001; elevation - F(12,108) = 37.72, p < 0.001), and a significant interaction of probe by target location (azimuth - F(12,108) = 2.66, p = 0.001; elevation - F(12,108) = 3.66, p < 0.001). Paired-samples t-tests showed a significant probe effect on the divergence of the systematic error field (t_9_ = 2.26, p = .050), which was smaller following the location than color probe (Figure S4A).

The systematic errors in CS and CM coordinates showed a significant effect of probe (azimuth - F(1,9) = 20.66, p = 0.001; elevation - F(1,9) = 8.54, p = 0.017), reference frame (azimuth - F(1,9) = 10.31, p = 0.011; elevation - F(1,9) = 4.10, p = 0.073) and probe by reference frame interaction (azimuth - F(1,9) = 16.55, p = 0.003; elevation - F(1,9) = 7.93, p = 0.020), the error in CM, but not CS, coordinates being much smaller following location than color probes (Figure S4B).
